# Disaster response among hospital nurses dispatched to evacuation centers after the Great East Japan Earthquake: a thematic analysis

**DOI:** 10.1186/s12913-022-08231-8

**Published:** 2022-07-01

**Authors:** Chika Yamamoto, Chieri Yamada, Katsuko Onoda, Morihito Takita, Yasuhiro Kotera, Arifumi Hasegawa, Tomoyoshi Oikawa, Masaharu Tsubokura

**Affiliations:** 1 Department of Emergency, Minamisoma Municipal General Hospital, Fukushima, Japan; 2 Department of Disaster and Radiation Medical Sciences, Fukushima Medical University, Fukushima, Japan; 3 Department of Public Health Nursing for International Radiation, Fukushima Medical University, Fukushima, Japan; 4 Department of Nursing, Minamisoma Municipal General Hospital, Fukushima, Japan; 5 Department of Radiation Health Management, Fukushima Medical University School of Medicine, Fukushima, Japan; 6 School of Health Sciences, University of Nottingham, Nottingham, UK; 7 Department of Radiation Disaster Medicine, Fukushima Medical University School of Medicine, Fukushima, Japan; 8 Department of Neurosurgery, Minamisoma Municipal General Hospital, Fukushima, Japan; 9 Research Center for Community Health, Minamisoma Municipal General Hospital, Fukushima, Japan

**Keywords:** The Great East Japan Earthquake, Nuclear power plant accident, Nurses, Victims, Disaster preparedness, Evacuation centers

## Abstract

**Background:**

Disaster relief operations involve a variety of components of healthcare efforts. The post-disaster recovery is a key component of hospital preparedness. This study aimed to investigate the role of hospital nurses in the disaster area and their challenges during the relief efforts after the Great East Japan Earthquake in 2011.

**Methods:**

Semi-structured interviews were conducted with ten nurses who worked in a general public hospital before the Great East Japan Earthquake and were dispatched to the evacuation centers after the disaster. A qualitative approach with the thematic analysis method was employed. Three research queries (RQs) were prepared before the interview.

**Results:**

The study participants played administrative roles as city employees in addition to performing nursing services as healthcare providers in evacuation centers. The first RQ on their challenges in evacuation centers gave us four themes: criticism by the evacuees, conflicts between multiple roles, difficulties in performing the first experience, and anxiety in working. The second RQ asking about motivation to accomplish disaster relief efforts raised three themes of carrying out the nursing role, acceptance by evacuees, and strengths of human connections. Two themes of awareness of disaster medicine and professional growth were raised from the third RQ of gains from the experiences in the evacuation centers.

**Conclusions:**

The hospital nurses in the disaster area performed multiple roles in the relief efforts in the evacuation centers, which developed a psychological burden on them. A sense of competence supported the motivation to accomplish the disaster relief activities and professional growth as a specialist in disaster medicine. A study limitation is missing hospital nurses who resigned during the relief efforts. Further study is warranted to refine the disaster preparedness of hospital operations.

**Supplementary Information:**

The online version contains supplementary material available at 10.1186/s12913-022-08231-8.

## Background

Disaster relief operations involve a variety of components of healthcare efforts. The World Health Organization (WHO) summarized the key elements of hospital operations for disaster preparedness [1]. The WHO’s checklist covers the medical triage of disaster victims, the command system for emergency management, human resources, capacities of medical care, and supply management. In the post-disaster recovery part, management of the hospital staff was listed in addition to the assessment of the facility, medical equipment, and supply. Previous studies on the hospital preparedness for natural disasters revealed the area to be enhanced, such as logistics of medical supplies, management of essential treatment, and process control [2, 3]. A meta-analysis containing 26 articles out of 1,545 studies on disaster preparedness found that the command control and post-disaster recovery were common issues for hospital preparedness [4]. In the case of an incidence following the mass gathering, the surge capacity was identified as a risk of the hospital operation [5, 6]. The healthcare failure mode and effect analysis (HFMEA) designed by the National Center for Patient Safety (NCPS) in the Department of Veterans Affairs in the United States is also helpful to proactively access the hospital preparedness to ensure the patient safety and quality of care [7]. An operational exercise has been highly recommended on a regular basis for disaster preparedness [8].

Healthcare in the evacuation center is a crucial component of disaster relief [9]. Multiple professionals inside and outside disaster areas are involved in managing evacuation centers [10, 11]. The Disaster Medical Assistance Team (DMAT) is a medical team consisting of healthcare professionals arriving from non-disaster areas to the disaster area [12, 13]. Their quick visit significantly helps alleviate the surge capacity of healthcare providers, which is a key component of the WHO’s checklist. Meanwhile, the role of healthcare staff in the disaster area is of importance since they know the characteristics of local patients and residents, geography for logistics, and medical resources. Previous reports have examined the types of support by medical professionals from outside the disaster area [14–16]. However, investigating the difficulties that the healthcare professionals in a disaster area face during their relief efforts is relevant to the recovery of local medical institutions.

**Fig. 1:**
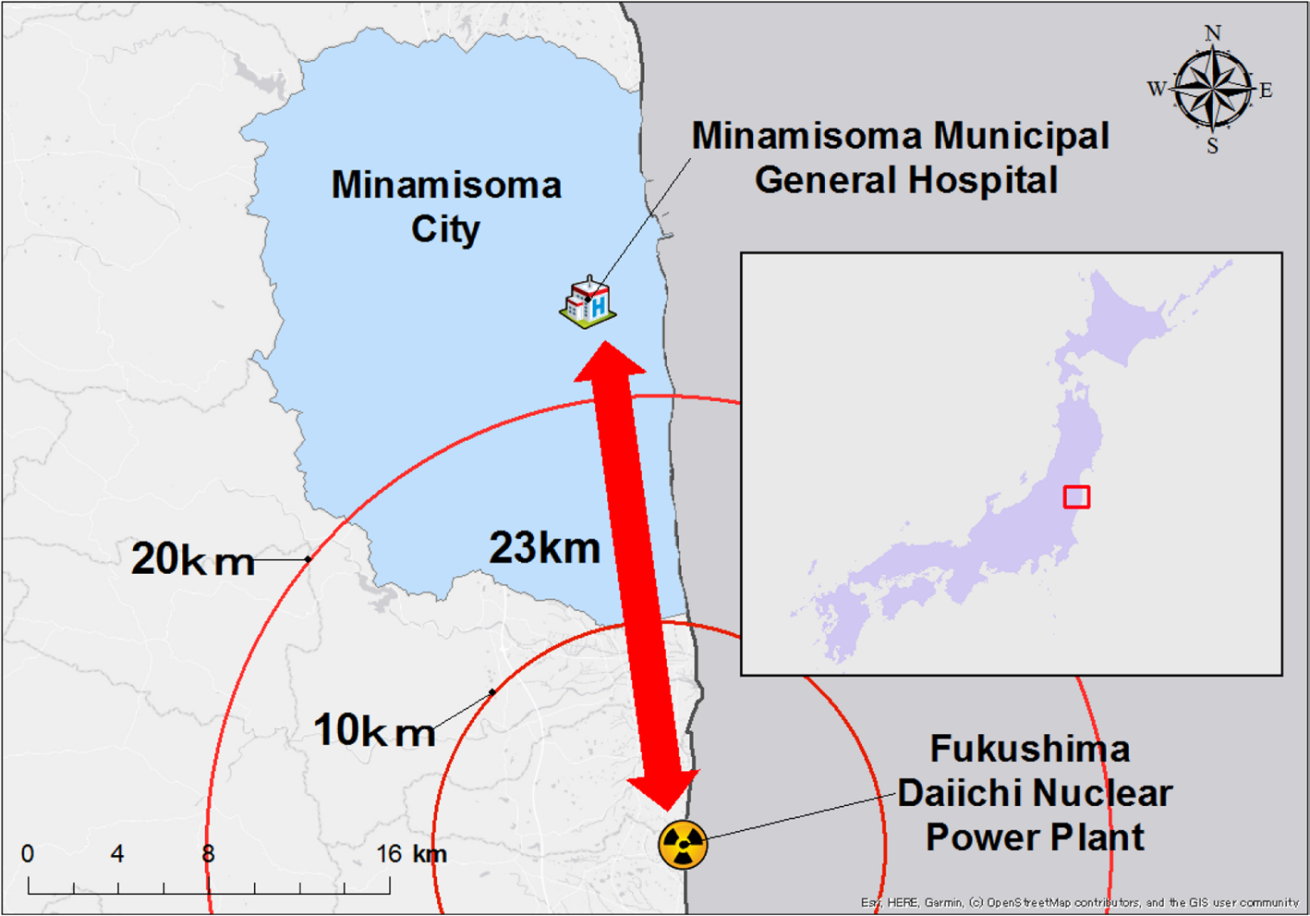
Geographical presentation of the Minamisoma Municipal General Hospital and the evacuation area after the disaster. On March 12, evacuation orders were issued to residents within a 20-km radius of the Fukushima nuclear power plant. On March 15, indoor evacuation orders were issued to residents within a 20- to 30-km radius

The Great East Japan Earthquake in March 2011 was followed by a huge tsunami and an accident at the Fukushima Daiichi Nuclear Power Plant (FDNPP) [17]. The triple disaster combined with a great earthquake, tsunami, and nuclear accident is unique in the history of the world. Minamisoma City in the Fukushima Prefecture, Japan, is located north of the FDNPP, within a 12–38 km radius. The Minamisoma citizens were forced to evacuate due to the FDNPP accident in addition to the damages caused by the earthquake and tsunami [18, 19]. In an unprecedented decision, the Minamisoma Municipal General Hospital (Fig. 1), located within 23 km of the FDNPP, dispatched its medical staff to evacuation centers where Minamisoma citizens moved, depending on their location willingness. This study aimed to investigate the role of nurses during the disaster relief efforts in complex situations and the challenges they faced. The finding in this study would suggest key elements of hospital preparedness in terms of post-disaster recovery.

## Methods

### Participants

The nurses who fulfilled the following conditions were recruited after the approval of the institutional review board and the director of the Minamisoma Municipal General Hospital Nursing Department; *i*) those who were working at the Minamisoma Municipal General Hospital at the time of the Great East Japan Earthquake, and *ii*) those were involved in supporting activities at the evacuation centers after the great earthquake. Semi-structured interviews were conducted with an interview guide (Appendix A).

### Research design

The thematic analysis of interview data was utilized in this study to identify the risks and challenges the participants faced during the disaster relief activities [20]. We followed the six steps proposed by Braun and Clarke [21].

### Research procedures and analysis

Prior to the interviews, all participants were given a set of pre-designed interview questions regarding shelter support activities (Appendix A). The interview included the following research questions (RQs) in addition to participant characteristics of years of nursing practice when the disaster occurred, gender and operations, and the transition in the disaster relief efforts;

RQ1: What were the difficulties that hospital nurses faced during the disaster relief efforts in the evacuation centers?

RQ2: What were the motivations to continue the relief efforts in the evacuation centers?

RQ3: What was learned from the experience during disaster relief efforts in the evacuation centers?

The first author, CY, interviewed all participants individually. The interviews were confidentially conducted in an independent space for approximately 60 min between March and May 2020. The interviews were recorded and transcribed verbatim. The records were analyzed using descriptive qualitative research methods.

Recordings were divided into meaningful segments and summarized for the categories and sub-categories based on the homogeneity and heterogeneity of semantic content. The interview data were carefully interpreted to generate the candidates of themes (*familiarization*). The coding was implemented to provide labels for the data in order to begin a systematic analysis of text data (*generating the initial codes*) [22]. Microsoft Excel was used to categorize and organize the themes against the individual RQs in the interview guide (*searching for themes*). Triangulation of the research queries was performed to ensure transparency and consistency (*reviewing themes*) [22]. For each identified theme, CY and MT checked the validity and reliability of the data extraction quality information, respectively. In case of any inconsistencies in coding, the authors discussed and reached a consensus on all themes. The nature and scope of the collated data were reviewed to ensure that each theme was presented to accurately illustrate the accompanying narratives (*definition and naming of themes*).

### Ethical considerations

All methods in this study were performed in accordance with Helsinki’s declarations and SROR (Standards for Reporting Qualitative Research). This study was approved by the Ethics Committee of the Minamisoma Municipal General Hospital (Approval number: 1–17) and the Ethics Committee of the Fukushima Medical University (Approval number: 2019–330). Written informed consent was obtained from all research participants.

## Results

### Characteristics of participants and their efforts after the disaster

Ten nurses, consisting of nine women and a man, were included in this study (Table 1). The median years of nursing experience at the time of the disaster was 11 (range: 5–20) years. The Great East Japan Earthquake was the first experience performing disaster relief operations, including working at evacuation centers, for all participants. There were three different circumstances before the assignment to work at the evacuation center; pattern *A*) those who served as a city employee just after moving to the evacuation center (*n* = 2), *B*) worked as a volunteer at the evacuation center for a while and then formally assigned as a city employee for disaster relief (*n* = 2), and *C*) continued working at the city hospital and then shifted to the evacuation centers (*n* = 6).

**Table1:** Participant characteristics

Participant	Years of nursing practice at the disaster (years)	Sex	Operations at disaster and their transition
pI	20	Female	Provided healthcare services and played the administrative roles in the evacuation center where she evacuated just after the disaster
pII	10	Female	Worked at the city hospital for a while, and then shifted to the evacuation centers
pIII	5	Man	Worked at the city hospital for a while, and then shifted to the evacuation centers
pIV	19	Female	Provided healthcare services and played the administrative roles in the evacuation center where she evacuated just after the disaster
pV	14	Female	Worked as a volunteer at the evacuation center where she evacuated for a while, and then, formally assigned as a city employee for disaster relief
pVI	13	Female	Worked at the city hospital for a while, and then shifted to the evacuation centers
pVII	10	Female	Worked at the city hospital for a while, and then shifted to the evacuation centers
pVIII	10	Female	Worked at the city hospital for a while, and then shifted to the evacuation centers
pIX	9	Female	Worked at the city hospital for a while, and then shifted to the evacuation centers
pX	19	Female	Worked as a volunteer at the evacuation center where she evacuated for a while, and then, formally assigned as a city employee for disaster relief

Participant characteristics and their transitions of efforts are shown

Their efforts after the disaster are summarized in Table 2. We classified their tasks into the five categories of assistance for healthcare for evacuees, assistance for medical care, arrangement of healthcare services with the other professionals, livelihood support, and administrative roles. The study participants played administrative roles as city employees in addition to performing nursing services as healthcare providers.

**Table 2:** Nurses’ efforts in the evacuation centers after the disaster

Category	Efforts
Healthcare for evacuees	Performing health checkup for the evacuees along with patrolling the evacuation centers with public health nurses and volunteer nurses
	Assessing oral conditions and teaching oral hygiene
	Managing medications for evacuees
	Screening the deep-vein thrombosis in the evacuees, and instructing them to use the compression stockings and perform the preventive exercises
	Explanation of health counseling at secondary evacuation centers and home-visit health counseling
	Hygiene management in evacuation centers
Assistance for medical care	Assistance for physician consultations
	Providing residents with information on the medical institutions
	Coordination of a medical examination
Arrangement of healthcare services with other professionals	Assessing the level of care required for the evacuees
	Request healthcare services to the public health nurses
	Arrangemeng of meetings with attending physicians, nurses, and clinical psychologists
	Listing up the subjects who needed the physical or occupational therapies, and sharing their information with the medical staff
Livelihood support	Promoting exercise to prevent physical weakness
	Providing a place for children to release their stress and engage them in preschool lives
	Assisting residents’ shopping trips
	Working with evacuees with a history of mental illness
Administrative roles	Identification of the evacuees with the records in the city office
	Explaining the situation of the city to the residents with administrative staff
	Responding to the applications for donations
	Explain the disaster certification to residents
	Organizing the documents for residents who have left the evacuation centers
	Responding the phone calls from residents regarding administrative matters
	Surveying residents who wish to return their home
	Notifing itinerary for those who wish to return home
	Sorting relief supplies
	Explanation of the applications for the temporary housing
	Survey of the citizens’ opinion
	Response to inquiries from the mass media of television and newspapers
	Preparing the venue for the city council members’ rounds, and attendance at their meetings

Nurses’ efforts in the evacuation centers after the disaster were summarized. Five categories were identified after the interpretation by authors

### Themes and the subthemes

The following themes and subthemes were identified after preparing a master codebook with 141 codes (Appendix B). The four themes with nine subthemes, three with seven, and two with seven, were generated for RQ1, RQ2, and RQ3, respectively. The main themes and the subthemes with representative codes were described in the following sections.

### RQ 1) Challenges in evacuation centers

Four themes were raised for RQ1: *i*) experiences of not being recognized as an evacuee by residents, *ii*) conflicts in multiple positions, *iii*) difficulties in performing tasks that they had never experienced before, and *iv*) anxiety in working (Table 3).

**Table 3:** Themes and subthemes of RQ 1: *Challenges in evacuation centers*

Themes	Subthemes	Examples of codes
Experiences of not being recognized as an evacuee by residents	Experiences of being hurt by residents’ words	Residents lost their jobs due to the disaster. Because we were working, the residents told us, “I envy you.” We were the outlet for their frustration. (pIV)
		It was hard to hear residents say, “We can’t do our jobs because we lost our jobs.” (pI)
		I was most shocked when a resident cursed me, saying, “You’re on the government side.” (pVI)
		Residents were confronted with stress caused by the disaster, dissatisfaction with the shelters, and a desire to return home. (pII, III, IV, and IX)
	Sadness at not being reconized as an evacuee	They just saw me as a public servant and did not see me as a disaster victim. (pI, II, VI, and IX)
Conflicts between multiple positions	Conflicts between roles at home and thoughts as a nurse	During the evacuation, I had conflicts between roles as a mother, a nurse, and a city employee. (pIV)
	Conflicts between being a city employee and a nurse	Residents identified us as both a nurse and a city employee. (pI, VI, and IX)
Difficulties in performing tasks which they had never experienced before	Difficulties in working in a field different from clinical practice	My role at the evacuation centers was public health service. I felt tha my knowledge was insufficient because it was different from nursing care in a hospital. (pII, VIII, and IX)
	The difficulty in the first experience	I had never been involved in disaster relief before, and this was my first time working in an evacuation center.(pII, IV, V, and VIII)
		It was my first time being affected by a major disaster and working in an evacuation center, so I was nervous the whole time. (pVI, and VII)
	Difficulties in making decisions when responding to the individual cases	While visiting a temporary housing complex, I found a resident collapsed inside. Since there was no standard of procedures for handling the situation, I was confused whether I should call an ambulance or not. My decision might cause messy conflict in the other evacuees. (pX)
Anxiety in working	Anxiety	We were also anxious about what would happen to us, and it was breaking our hearts. (pIV)
	Uncertainty in losing the goal	All I could think about was how I was going to spend my time and deal with it on a daily basis. (pVI)
		It was all I could do to spend each day. (pV)

The main theme of *experiences of not being accepted by residents* included two subthemes; *RQ1-i-a*) experiences of being hurt by residents’ words and *RQ1-i-b*) sadness at not being recognized as an evacuee. Participants were surrounded by residents who had lost their jobs as a result of the disaster when they visited the evacuation centers. The study participants mentioned their emotional stress caused by the disaster, the following evacuation, dissatisfaction with life in the shelter, and their experiences of being hurt by the residents’ words.

“Residents lost their jobs due to the disaster. Because we were working, the residents told us that they envy us. We seemed to become the outlet for their frustration.” (pIV).

“They just saw me as a public servant and did not see me as a disaster victim.” (pVI).

“Residents were confronted with stress caused by the disaster, dissatisfaction with the shelters, and a desire to return home.” (pII).

They spoke of their sadness that they were not recognized as evacuees even though they had experienced the same disaster.

“The residents just saw me as a public servant and did not see me as a disaster victim.” (pI).

The second theme of *RQ1*, which is the conflicts between multiple positions, contained two subthemes; *RQ1-ii-a*) conflicts between roles at home and thoughts as a nurse, and *RQ1-ii-b*) conflicts between being a city employee and a nurse.

“During the evacuation, I had conflicts between roles as a mother, a nurse, and a city employee.” (pIV).

“Residents identified us as both a nurse and a city employee.” (pIX).

It is clear that they were engaged in disaster relief activities in the evacuation centers while they had feelings of the responsibilities of a mother, a nurse, and a city employee. In addition to this, the subtheme of *sadness at not being recognized as an evacuee (RQ1-i-b)* shows the conflict of feelings between the disaster victims and staff members for disaster relief.

The third thems of *RQ1*, which is the *difficulties in performing tasks that they had never experienced before*, included three subthemes; *RQ1-iii-a*) difficulties in working in a field different from clinical practice, *RQ1-iii-b*) difficulty in the *first* experience, and *RQ1-iii-c*) difficulties in making decisions when responding to individual cases.

“My role at the evacuation centers was public health service. I felt that my knowledge was insufficient because it was different from nursing care in a hospital.” (pVIII).

The participants commented on the difficulty of working in a field different from clinical practice, where public health services and administrative tasks as city employees are required to perform. Many of the participants also commented on the difficulty of their first experience in disaster relief activities in the evacuation centers.

“It was my first time to be affected by a major disaster and to work in an evacuation center, so I was nervous the whole time.” (pVII).

In addition, there were comments on the difficulties in making decisions when responding to individual cases. A participant described her experience of being perplexed by the burden of calling an ambulance when she had not yet established a relationship with the evacuees in the temporary housing. This was because she was not able to confirm the will of the residents fully.

“While visiting a temporary housing complex, I found a resident collapsed inside. Since there was no standard of procedures for handling the situation, I was confused about whether I should call an ambulance or not. My decision might cause messy conflict in the other evacuees.” (pX).

The fourth theme of *RQ1*, which is the *anxiety in working*, included two subthemes; *RQ1-iv-a*) anxiety and *RQ1-iv-b*) uncertainty in losing the goal. The participants were faced with uncertain choices when they started their activities at the evacuation center, and it is evident that they were anxious about the future of their families and themselves.

“We were also anxious about what would happen to us, and it was breaking our hearts.” (pI).

“All I could think about was how I was going to spend my time and deal with it on a daily basis.” (pVI).

“It was all I could do to spend each day.” (pV).

### RQ2) Motivations to continue the disaster relief efforts in the evacuation centers

Three themes were raised for *RQ2*; *i*) carrying out the role of a nurse, *ii*) acceptance by residents, and *iii*) strengths of human connections (Table 4). The first theme of *RQ2*, which is the *carrying out the role of a nurse*, included two subthemes; *RQ2-i-a)* the participants described their rewarding experiences as a nurse by providing healthcare services in evacuation centers, and *RQ2-i-b)* their experiences in communicating with residents, listening to their thoughts and feelings, identifying their needs, and providing care to meet those needs.

**Table 4:** Themes and subthemes of RQ 2: *Motivations to continue the disaster relief efforts in the evacuation centers*

Themes	Subthemes	Examples of codes
Carrying out the role of a nurse	Rewarding	I thought that I did help improve the evacuees’ health conditions by providing nursing care. (pI and III)
	Providing healthcare services that met the needs of evacuees	In order to alleviate residents’ concerns as much as possible, all we can do was to listen to them in an accommodating manner (pIII, IV, and VII)
		Evacuees just needed their listeners. (pI, II, V, VI, and X)
Acceptance by residents	Build a trusting relationship with residents	I was very happy when someone said, “I can talk to you easily because you are a nurse from my hometown and I can understand your dialect. (pI and IV)
	Reduce residents’ anxiety	Some residents said, “Because we are from the same hometown, we can talk to each other and feel at ease.” (pII)
Strengths of human connections	Strengths of having a family together	My family members were with me, so I felt a little reassured (pI)
	Strength of being with friends	I was helped by the staff who were working together (pII)
		I felt more comfortable when I worked my teammates than when I was alone (pIII and IV)
	The support of people in the shelter community	I have many good memories, such as festivals at evacuation centers (pII, VII, and VIII)
		Evacuees did not mind the radiation accident. They accepted us and were kind and concerned about us (pI and III)
		I evacuated to the area that suffered by the Chuetsu Earthquake in 2007. The residents were kind enough to say to us, “You are also victims of the earthquake, so please try to take care of yourselves emotionally”. (pIII and VII)

“I thought that I did help improving the evacuees’ health condition by providing the nursing care.” (pIII).

“In order to alleviate residents’ concerns as much as possible, all we can do was to listen to them in an accommodating manner.” (pIV).

“Evacuees just needed to their listeners.” (pX).

In the second theme of *RQ2*, which is the *acceptance by residents*, two subthemes were raised; *RQ2-ii-a*) build a trusting relationship with residents, and *RQ2-ii-b)* reduce residents’ anxiety. Despite the harsh words from the residents at the beginning of the disaster relief activities, the participants shared their experiences in the evacuation centers with evacuees from the same hometown. They could alleviate the residents’ anxiety and build a trusting relationship with them.

“I was very happy when someone said that they can talk to me easily because I am a nurse from their hometown and they can understand my dialect.” (pI).

“Some residents said, because we are from the same hometown, we can talk to each other and feel at ease.” (pII).

The third theme of *RQ2*, which is the *strengths of human connections*, included three subthemes; *RQ2-iii-a*) strengths of having a family together, *RQ2-iii-b*) strength of being with friends, and *RQ2-iii-c*) support of people in the shelter community. The presence of their own family nearby the evacuation centers provided emotional support. In addition, the participants mentioned the presence of their friends with whom they had shared disaster relief efforts. They commented that assistance by the residents who had set up the shelters was also a source of motivation.

“My family members were with me, so I felt a little reassured” (pI).

“I felt more comfortable when I worked my teammates than when I was alone.” (pIV).

“Evacuees did not mind the radiation accident. They accepted us and were kind and concerned about us” (pIII).

“I evacuated to the area suffered by the Chuetsu Earthquake in 2007. They were kind enough to say to us, “You are also victims of the earthquake, so please try to take care of yourselves emotionally”.” (pVII).

### RQ3) Gains from experience at the evacuation centers

Two themes were raised for RQ3: *the gains from experience*; i) increased awareness of disasters, and *ii*) growth as a nurse (Table 5). A theme of RQ3 of the *increased awareness of disasters* includes four subthemes; *RQ3-i-a*) opportunity for disaster nursing, *RQ3-i-b*) preparation for disaster, *RQ3-i-c*) need to pass on experience, and *RQ3-i-d*) change to a positive attitude.

**Table 5:** Themes and subthemes of RQ 3: *Gains from experience at the evacuation centers*

Themes	Subthemes	Examples of codes
Increased awareness of disasters	Opportunity for disaster nursing	I got a certificate as a member of the disaster medical assistance team (DMAT) after the earthquake (pIII and IV)
		After the earthquake, I registered as a nurse specializing in disaster relief (pVIII)
		I always want to help someone with difficulties (pIV and IX)
		I want to be a person who can do the same thing as the people in the evacuation centers who supported us. (pI and III)
	Preparation for disaster	I think about how I can help when disaster strikes my location (pIV and V)
		I always make sure that my family is prepared for any disaster, and I make use of the lessons I learned from that time, such as being out of water. (pVI and IX)
	Need to pass on experience	I participated in disaster relief after the Kumamoto earthquake, and I definitely made use of my experience in 2011. (pIV)
		I hope we can pass on our experiences to others. We have no choice but to make use of the lessons we have learned(pIIIVI)
	Change to a positive attitude	I am here now because of my various experiences (pVII and VIII)
		I started to think positively (pV)
		Despite difficulties, this experience turned out to be a blessing in disguise (pIII and IV)
Growth as a nurse	Renewed awareness of responsibility as a nurse	The feeling that the work must be done responsibly. (pV, VI, and IX)
	Reaffirming that a public health effort is an object of nursing	I thought that the target audience for nursing was not only people in hospitals but also all residents when considered from the perspective of health. (pVI)
		It has changed my feelings about my job and about being allowed to do nursing. (pII and III)
		It broadened my perspective as a nurse (pIV and V)
	Changes in relationships with people	What has changed since the disaster is that I have more interaction with people in the community. (pVI and X)
		I felt the importance of communication again. (pIII)

The participants talked about how their experiences in the disaster and in assisting people in evacuation shelters made them aware of disaster relief activities. Their experience promoted them to involve in disaster relief for another disaster. They also told how they could apply their experiences in the great disaster in 2011 to the future disaster. They highlighted the need to pass the lessons learned from their disaster relief experiences to the next generation of nurses. In addition, they talked about how they prepare daily for future disasters. They felt that any aspects of their experiences at the disaster, including the painful experiences, have helped their personal growth.

“After the earthquake, I registered as a nurse specialized for disaster relief” (pVIII).

“I always make sure that my family is prepared for any disasters, and I make use of the lessons I learned from the disaster in 2011, such as not running out of water.” (pIX).

“I hope that we can pass our experiences to other nurses. We have no choice but to make use of the lessons we have learned” (pVI).

“I am here now because of my various experiences.” (pVII).

The second theme of *RQ3*, which is the growth as a nurse, includes two subthemes; *RQ3-ii-a*) renewed awareness of responsibility as a nurse, *RQ3-ii-b*) reaffirming that the public health effort is an object of nursing, and *RQ3-ii-c*) changes in relationships with people. The participants who could not attend the disaster relief efforts in the city hospital just after the disaster expressed their regrets about choosing to evacuate to the remote area.

“The feeling that the work must be done responsibly.” (pV).

Many commented that the support activities at the evacuation centers helped them grow as nurses. They spoke about their experiences of having to make choices about evacuation during the disaster, experiencing the deaths of many people close to them and many others, and their involvement with residents, which led them to a renewed awareness of their responsibilities as nurses. In addition, while conducting health activities for residents at evacuation centers, she was given an opportunity to reaffirm that the target of nursing is the residents. They talked about how they overcame the confusion of being affected by the same disaster as the local residents and how they continue to have relationships with the residents to this day.

“I thought that the target for nursing care was not only patients in medical institutions but also all residents when I considered from the view of public health.” (pVI).

“My feelings about my job have changed as an appreciation for working as a nurse.” (pII).

“What has changed since the disaster is that I have more interaction with people in the community.” (pX).

These narratives suggest that their experiences had a significant impact on their current nursing services, as they felt that their experiences then were experiences that led to the present and led to their own personal growth.

## Discussion

This study revealed the challenges, perceptions, and learning gains of the hospital nurses who worked at the medical institution close to the FDNPP and were involved in the disaster relief effort in the evacuation centers after the Great East Japan Earthquake in 2011. Their experiences are particularly important for *disaster nursing* since the disaster in Fukushima, Japan, in 2011 was a unique and complex event consisting of a great earthquake, huge tsunamis, and a nuclear power plant accident. We identified the key elements of their experiences using a qualitative approach of theme analysis which suggests disaster preparedness for a medical institution.

### Challenges in disaster relief efforts in evacuation centers

During the disaster relief efforts, a challenge was the emotional stress of working and being a disaster victim. While the nurses were evacuees of the earthquake, tsunami, and nuclear accident, they were also the city employees. There was confusion, as we can see from the comment: “the residents just saw me as a public servant and did not see me as a disaster victim,” at the beginning of their efforts. The nurses had conflicting feelings regarding their roles as city employees and disaster victims. In such a situation, the words of blame from the residents put more emotional stress on the nurses.

The experience of being hurt by the words of residents has also been reported by the local government officers and firefighters after the Great East Japan Earthquake [23–25]. The FDNPP accident developed the condemnation to the public officials by residents. More than half of the local government employees affected by the nuclear power plant reported that the communication with residents was stressful for their work [26].

The eruption at Miyakejima island, Japan, in 2000 was a disaster that resulted in the mass evacuation of residents. Takamatsu reported on her nursing activities as an employee of the clinic during the Miyakejima eruption, but her report did not mention the emotional stress [27, 28]. Fujino compared the evacuation after the Miyakejima eruption with that after the FDNPP accident [29]. In the case of Miyakejima, the victims had a sense of resignation because it was a natural disaster. However, in Fukushima’s case, the FDNPP accident led to criticism of the government, which had promoted the nuclear power project. The Fukushima residents were under emotional stress due to the evacuation, fear of radiation exposure, and uncertainty about the future. In turn, the local government officers were to blame by the evacuees. The criticism from the residents to the nurses in this study may have originated from such a complex background. In addition to medical assistance immediately after the disaster, mental care should be provided to the evacuees. At the same time, it is important to establish emotional support for nurses, the other healthcare providers, and the local government officers even immediately after the disaster.

### Motivations to continue the disaster relief efforts in the evacuation centers

In this study, the nurses’ strong will early after the disaster was revealed to fulfill their tasks as healthcare providers and city employees as well. Later, they were motivated by the kind words from the residents to continue the disaster relief efforts.

Previous studies showed that nurses were expected to have an empathetic understanding of patients [30]. An attitude of listening is required for the mental care at the evacuation centers. When listening to the evacuees, their needs are expected to be identified, which leading a sense of fulfillment as a nurse [31]. The nurses shall understand their needs and make provisions accordingly. Such interactions with evacuees might develop the nurses’ confidence and their will to continue working.

Nursing care in a hospital is different from that for the community as a public healthcare service. However, there might be a common nursing principle between clinical and community health management [32]. The home-visiting nursing care can connect the two different fields. We believe that the close communication between nurses working in a core hospital and those in the community as home-visiting care providers would enhance disaster relief efforts.

The situation that the evacuees and the nurses in the same city of *Minamisoma* evacuated to the same evacuation center might be beneficial to solving the emotional discrepancies between the evacuees and the nurses. The kind words to nurses from the evacuees became an experience of “being accepted by the residents” and “emotional support.” This can also be evident in the words of the “great presence of friends” mentioned in “Strengths of ties with residents.” The trust relationship between the hospital staff and the residents during the normal time would promote stress-coping behavior in a crisis time of disaster.

Trauma-informed care (TIC) might be helpful for traumatic experiences. In TIC, the healthcare providers understand the nature of the trauma and tell the subjects and their surroundings appropriate coping strategies [33, 34]. Although we do not know whether or not the participants of our study were aware of the term ‘*TIC*,’ they were able to recognize it accurately by confronting traumatic events, such as being blamed by residents and were able to talk to each other about their feelings during their disaster relief activities. In this way, disaster relief workers should have opportunities to learn about the traumatic disorder and how to cope with it. Understanding traumatic disorders could enhance disaster relief efforts.

### Gains from experience at the evacuation centers

The experience in the evacuation centers promoted awareness of disaster preparedness and their profession as a nurse. When a disaster strikes, the operations in the medical field are in turmoil, resulting in an environment with a high mental burden. Under such conditions, medical professionals, not just nurses, are forced to operate in confusion, anxiety, and fear, but also with a sense of mission. However, in this study, it became clear that such difficult situations were not only negatively influenced but also gave the nurse an opportunity for professional growth, such as the code of “growth as a nurse.” Our finding is consistent with the previous studies showing post-traumatic growth [35–37]. The professional growth will be helpful for future disaster relief and medical crises such as the pandemic of the severe acute respiratory syndrome coronavirus-2 (SARS-CoV-2). Common issues between the disaster relief efforts after the great earthquake and the response to the SARS-CoV-2 pandemic are difficulties in performing tasks that the healthcare providers had never experienced before and anxiety in working. Their experience in evacuation centers can maintain the motivation of nurses to overcome difficulties in clinical settings.

### Limitations of this study

This study has several limitations. First, the qualitative nature of the study limits the generalization of the study’s findings. The disaster combined with the earthquake, tsunami and nuclear accident also limit applying our findings to other conditions of disaster. Second, we missed the hospital nurses who resigned during and after the disaster relief efforts. Thus, we might not sufficiently analyze the challenges which the hospital nurses faced in evacuation centers. Third, since the interviews were conducted nine years after the disaster, some of the subjects might not accurately recall the disaster at the time of the interviews. Thus, the recall bias might exist.

## Conclusion

The present study with a qualitative approach revealed the challenges for the hospital nurses when they performed the disaster relief efforts in the evacuation centers after the Great East Japan Earthquake in 2011. Their challenges included the conflicts between multiple roles as a healthcare provider, a city employee, and a disaster victim. The tasks in the evacuation centers that they had never been trained in before the disaster might develop a psychological burden on them. A sense of competence supported the motivation to accomplish the disaster relief activities and professional growth as a specialist in disaster medicine. A limitation of this study is that we missed the hospital nurses who resigned during the relief efforts, which will be a scope of future study. Our study contributes to refining the disaster preparedness of hospital operations.

## Supplementary Information

**Additional file 1.** Appendix A

**Additional file 2.** Appendix B

## Data Availability

The dataset analyzed in this study is not publicly available due to the subjects’ permission to use it only for data analysis and not for any other use, but it is available from the corresponding author upon reasonable request.
