# Supplementary material for: Disaster response among hospital nurses dispatched to evacuation centers after the Great East Japan Earthquake: a thematic analysis
Source: BMC Health Serv Res. 2022 Jul 1;22:848. doi: 10.1186/s12913-022-08231-8 (PMC9247954; doi:10.1186/s12913-022-08231-8)
Supplement: Supplementary file 1 — Additional file 1. Appendix A [file 12913_2022_8231_MOESM1_ESM.docx]

# Appendix A. Interview Guide

The Great East Japan Earthquake, followed by the Fukushima Daiichi Nuclear Power Plant accident, forced the residents to evacuate. As employees of a public city hospital in Minamisoma, the hospital nurses were dispatched to evacuation centers in and outside of Fukushima Prefecture. While there have been many reports of outside medical teams assisting in the aftermath of the nuclear accident, there have been no studies on the nurses of local core hospitals assisting in evacuation centers in the aftermath of the disaster. There is a possibility that disaster relief activities under similar conditions will be conducted in the future. Therefore, this study aims to clarify the actual situation of disaster relief efforts by the hospital nurses in the evacuation centers in the midst of chaos due to the great disaster. What this study reveals may also provide suggestions on how to respond to future disasters and how to prepare to disasters in normal times.

1. How many years of experience as a nurse did you have at the time of the disaster?

2. What kind of efforts did you make in the evacuation centers where Minamisoma citizens were evacuated? Did you encounter any difficulties during your activities in the evacuation centers? If so, what were the difficulties?

3. Did you encounter any difficulties during your activities at the evacuation center? If so, what were the difficulties?

4. What supported you to continue your activities in the evacuation centers?

5. How does your experience with the shelter activities relate to your current involvement as a nurse? What are they?
