# Supplementary material for: Disaster response among hospital nurses dispatched to evacuation centers after the Great East Japan Earthquake: a thematic analysis
Source: BMC Health Serv Res. 2022 Jul 1;22:848. doi: 10.1186/s12913-022-08231-8 (PMC9247954; doi:10.1186/s12913-022-08231-8)
Supplement: Supplementary file 2 — Additional file 2. Appendix B [file 12913_2022_8231_MOESM2_ESM.docx]

# Appendix B. List of codes generated (*n* =141)

Explosion

Radiation Hazards

Fear

We have no choice but to nurse

Effects on the human body

Evacuated with nothing

Unclear impact

Choice of family or work

Children

Only you can protect your children

Running away

Conflict

Abandonment of nursing work

Feelings of apology

Guilt about evacuation

Regret for evacuating without patients

Choice to evacuate

Feelings of having to stay in the hospital

Lifelong regret

It was not the evacuation I wanted

I would die if I stayed in the hospital

I acted with my children in mind

Responsibility for my position

My house was half destroyed

Safer in the hospital

Safety of children

Ensuring the safety of the family

Patient care

Continuing aftershocks

Participation in hospital continuity

Feeling unstable

Anxiety about not being able to contact family

Sense of responsibility

Fear of second explosion

Nursing care for patients

Inability to leave the hospital

The thought that I must stay

Lack of hospital staff

Decision to return to hospital from evacuation site

Worked for a while before making a choice

No gasoline, no evacuation

I didn’t think too much about it

I had to do what I had to do

Can’t give up on patients

A sense of duty as a nurse

Respect for each choice

Questioning the job description

Sorting supplies

Pride as a nurse

Alleviation of loneliness

I have no choice but to be a nurse

Have no choice but to follow the hospital’s instructions

As a city employee, protecting the health of the citizens

Determination that I may not be able to come back

Decisions based on a sense of responsibility

Supporting evacuation centers is also my job

Prepared to go to any evacuation center

Supporting evacuation centers while evacuating

Missing family members

Hospital director told me that I could freely choose to evacuate.

Was told by colleagues that I could evacuate because my children were small.

I was helping out at the evacuation site

I called the hospital because I knew I could not stay at the evacuation site

Residents saw me as a city employee

Worried about ourselves

Residents are also anxious

Residents who lost their jobs due to the disaster

jealousy of continued employment

From the residents’ point of view, nurses are also government employees

I am a victim of the disaster as well

I am blamed because I am a government employee

I was cursed for being a government employee

The biggest shock

Stress caused by the evacuation

Complaints and dissatisfaction from residents

Separate it as a job

Residents’ complaints were accepted.

I have no regrets about having provided support to the evacuation center.

The hardest thing for me was to vent my frustration even though we are the same disaster victims.

They don’t see us as the same victims.

Anxiety about being away from their families

Accumulation of stress among the children

My heart was breaking.

Spending every day is all I can do

I was afraid they would complain again next time.

Nervous all the time because it was my first time to support the earthquake and the evacuation center

I didn’t know what would happen to me.

It was better to do support activities than to be anxious and do nothing.

I am glad there was something I could do.

Nursing can play a role in the health care of disaster victims.

Residents want to be listened to.

Differences in thinking between nurses and public health nurses

Unfamiliar work

Respond to residents in a courteous manner

Listening and giving advice to the extent possible

Serious subjects from residents

Everyone is a victim of the disaster

Be there for evacuee residents

Visit as many places as possible to build relationships with residents

Glad to hear residents’ words

Same local residents and nurses

the value of our existence

Hot meals

we were once told we were not needed.

Resident’s words that we can consult with you because you are from the same community

the same hometown

Residents’ reassurance of being in the same hometown

The security of being with family

Enjoyed doing support activities

I was helped by the members I went with

I was able to relax by being with my friends

The warmth of the people in the host community

I was happy to be accepted at the evacuation site.

The people at the host community were kind and concerned about me.

Events held at various evacuation centers

Words of encouragement for ourselves

Lack of coordination of shelter support locations

Doubts about the attitude of the residents

Lack of knowledge

Wanting to be useful in some way

Increased awareness of disasters

How and what to do in case of disaster

Registration for DMAT

Learning lessons from the disaster and making the most of them

Disaster preparedness

Sense of responsibility as nurses

When another disaster occurs, we will protect our own patients in the hospital

Want to protect patients as nurses while protecting their families

Importance of communication

Increased interaction with people in the community after the disaster

The weight and preciousness of life

Lost a friend in the disaster

Expanding my horizons as a nurse

The target of nursing is all residents

We have no choice but to make the most of the earthquake experience

Lessons learned

The experience of supporting evacuation centers was positive for me

Various experiences lead to the present

There were hard times, but now everything is food for thought

The experience of the disaster has made me stronger.

I hope I can pass on my experiences.
